# *Lavandula angustifolia* Essential Oils as Effective Enhancers of Fluconazole Antifungal Activity against *Candida albicans*

**DOI:** 10.3390/molecules28031176

**Published:** 2023-01-25

**Authors:** Michalina Adaszyńska-Skwirzyńska, Małgorzata Dzięcioł, Danuta Szczerbińska

**Affiliations:** 1 Faculty of Biotechnology and Animal Husbandry, Department of Monogastric Animal Sciences, West Pomeranian University of Technology in Szczecin, Janickiego Str. 29, 71-270 Szczecin, Poland; 2 Faculty of Chemical Technology and Engineering, Department of Chemical Organic Technology and Polymeric Materials, West Pomeranian University of Technology in Szczecin, Piastów Ave. 42, 71-065 Szczecin, Poland

**Keywords:** lavender essential oil, linalool, fluconazole, anticandidal activity, antifungal activity, synergism

## Abstract

The increasing prevalence of *Candida albicans* resistance to commercial antifungal agents in recent decades has prompted modern medicine and veterinary medicine to search for combined treatment options. The aim of the study was to determine the activity of essential oils from different cultivars and morphological parts of the medicinal lavender (*Lavandula angustifolia*) in combination with fluconazole against *Candida albicans* ATCC 10231 strain. The effect of the combination of lavender essential oil with fluconazole was tested using the checkerboard method, and the obtained results were interpreted on the basis of fractional inhibitory concentration indices (FICIs). A synergistic interaction was found for all combinations of fluconazole with essential oils isolated both from flowers and leafy stalks of two tested lavender cultivars: ‘Blue River’ and ‘Ellagance Purple’. The observed enhancement effect of fluconazole antifungal activity was significantly stronger in the case of essential oils obtained from flowers and leafy stalks of ‘Blue River’ cultivar. Analogous studies were performed for linalool, one of the main components of lavender essential oils, and a similar synergistic interaction with fluconazole was found.

## 1. Introduction

Until recently, yeasts of the genus *Candida* were regarded as saprophytic organisms normally found in the animal and human body, rarely causing serious diseases. However, there has been a continuous increase in systemic fungal infections over the past 30 years. The problem is the excessive use of chemotherapeutic agents with a broad spectrum of activity both in treatment and in prevention [1,2,3]. Therefore, drug-resistant strains are increasingly often isolated or classified as species that have previously been rarely identified as etiological infection factors. As a result, there is an increase in mortality due to fungal infections [3].

Polyene antibiotics or azoles are most commonly used to treat acute yeast infection. Azoles show high fungicidal activity; fluconazole belongs to the third generation of drugs in this group, and it is often the drug of choice due to low toxicity to the host organism. Although fluconazole is considered to be one of the most effective antifungal drugs, there are nevertheless reports indicating an increasing frequency of isolation of resistant strains. This is the reason justifying the search for substances that could enhance its therapeutic effect. In vitro, fluconazole exhibits antifungal activity against the most clinically common *Candida* species (including *C. albicans*, *C. parapsilosis*, and *C. tropicalis*). The group of triazole derivatives, which includes fluconazole, is less hepatotoxic (liver damage) than imidazole derivatives (e.g., clotrimazole and miconazole). After oral administration, fluconazole is well absorbed, and its plasma levels (and overall bioavailability) reach over 90% of the concentration values observed after intravenous administration [4]. The antifungal activity of fluconazole is closely related to the structure of the fungal cell membrane. Underneath the multilayer cell wall, consisting mainly of mannoproteins and beta-glucan interlaced with chitin, there is a cell membrane made of, among others, ergosterol, a lipid component analogous to cholesterol found in animal cells. The mechanism of the antifungal activity of fluconazole and other azoles is based on the inhibition of ergosterol biosynthesis through the inhibition of lanosterol 14-α-demethylase, the enzyme responsible for converting lanosterol to ergosterol, which leads to an increase in the level of toxic 14-methylsterols, considered a source of azole antifungal activity. Inhibition of ergosterol synthesis results in disturbances in the structure of the cell membrane, growth inhibition, and changes in its permeability and function, leading in turn to the destruction of the fungal cell. At the same time, due to differences in the structure of ergosterol and cholesterol, substances that interfere with ergosterol biosynthesis do not cross-react with host cells [5]. 

In recent years, there has been an increasing number of reports in the literature regarding the low efficacy of azoles due to the phenomenon of acquiring resistance after long-term therapy with their use [1,6,7]. The genus *Candida* has developed a number of azole antifungal resistance mechanisms, including modification of the target enzyme, reducing the drug access to the target by activating multidrug efflux transporters, or a combination of these mechanisms. High values of MIC (minimal inhibitory concentration) are characteristic for fluconazole tested against fungal strains that developed these resistance mechanisms, which affects its in vitro, in vivo, and clinical efficacy [8]. Overexpression of genes encoding enzymes involved in ergosterol biosynthesis and mutations within these genes, resulting in amino-acid sequence changes, as well as overexpression of genes encoding drug transporters and mutations within the transcription factors regulating the transcription of these genes, plays an important role in *Candida* yeast resistance to azoles [3,9,10,11]. The mechanisms of antimicrobial resistance are classified as either primary or acquired and are related to specific or acquired characteristics of the pathogens. Genome variability (plasticity) is one of the important adaptation mechanisms to environmental conditions, such as host response or the action of an antifungal chemotherapeutic agent [12].

The problem of the shortage of effective yet safe drugs indicates the need to search for new active antifungal compounds to which microorganisms have not developed resistance. Nature is an invaluable source of products or compounds with antimicrobial properties [12,13]. Currently, a “return to nature” is being observed, expressed in the search for safe natural substances as potential, promising therapeutics in the prevention and treatment of fungal diseases and infections [14]. The literature suggests that essential oils, including lavender oil, can be an effective alternative to chemotherapeutics when controlling fungal infections, particularly candidiasis [13,15]. The mechanism of action of essential oils on microorganisms has not been fully understood, but their diverse chemical composition results in a wide range of biological activity and significant antifungal properties. Essential oils are rich in a wide range of volatile compounds such as terpenes and terpenoids (including aldehydes, ketones, alcohols, and esters), which determine their biological properties [16]. The antimicrobial effects of essential oils have been empirically confirmed in many studies, but the mode of action has not been fully elucidated [14,17,18,19,20]. It is known that the mechanisms of activity of essential oils against fungal cells are partly shared with those against bacteria. Some components of essential oils cause depolarization of the cell membrane, lowering the transmembrane potential, while affecting the function of membrane pumps that transport calcium ions and ion channels. A reduced pH gradient prevents ATP synthase function, thereby reducing intracellular ATP content [21]. Chen et al. [22] found that dill seed essential oil caused mitochondrial dysfunction in *C. albicans*. These authors showed that the oil damaged the membrane and decreased the amount of ergosterol in the membrane, which in turn increased its permeability and, consequently, led to the leakage of cytoplasmic content. The effect of tea tree oil on yeast membrane damage was demonstrated in a study of Cox et al. [23].

New strategies for the simultaneous application of essential oils with synthetic antifungal drugs are also proposed to enhance the therapeutic effect of such treatments. The application of essential oils in combination with chemotherapeutic agents is a promising line of research [14,19,24,25]. The advantage of such a combination is the fact that both chemotherapeutic agents and some of the essential oils are approved for use in humans and animals, in contrast to newly synthesized chemical compounds, which require long-term research and large financial outlays before approval as therapeutic agents. Furthermore, excessive use of popular antimicrobial drugs often leads to the development of resistance by the microorganisms. This problem is also actual in the case of fluconazole, which is considered as relatively save among other currently used antifungal drugs and, therefore, commonly used. Combined therapy with compounds of natural origin, including essential oils, may result in a significant reduction in *C. albicans* resistance development to azoles, including fluconazole. In addition, very low concentrations of natural additives which enhance antifungal activity of fluconazole can significantly reduce the MIC values and effective therapeutic doses of this drug. As result, humans or animals would experience fewer side-effects associated with high doses of synthetic drugs. Lastly, both economical costs and the negative environmental impact of such modern combined therapies can be also significantly lower in comparison to the conventional ones.

From a clinical point of view, comparative studies which determine the sensitivity of fungi to essential oils, in relation to the susceptibility of these microorganisms to standard antifungal drugs, as well as the possibility of enhancing the effects of drugs by their combinations with essential oils, are valuable. The aim of this study was to determine the effect of essential oils obtained from two different cultivars and various morphological parts of the medicinal lavender (*Lavandula angustifolia*), in combination with fluconazole against a reference strain of *C. albicans*. Additionally, similar comparative studies were conducted for one of the main components of lavender essential oil—linalool. Evaluation of the interactions of studied essential oils with fluconazole against the *C. albicans* ATCC 10231 strain and comparison of activity of essential oils obtained from two lavender cultivars (‘Blue River’ and ‘Ellagance Purple’) and their various morphological parts (flowers and leafy stalks) represent the novelty of this study.

## 2. Results

**Figure 1:**
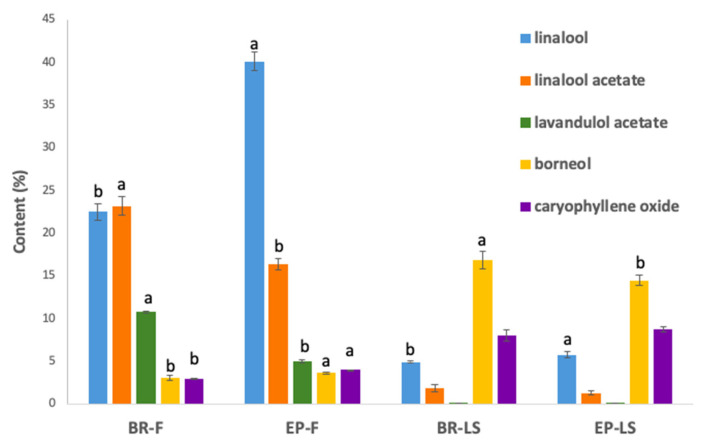
The main components of essential oils from lavender flowers (F) and leafy stalks (LS) of ‘Blue River’ (BR) and ‘Ellagance Purple’ (EP) cultivars (a, b—values with different letters differ significantly in flowers or leafy stalks among two different lavender cultivars at *p* < 0.05).

Table 1 presents the results of research on the effect of essential oils isolated from flowers and leafy stalks of two cultivars of *L. angustifolia* (‘Blue River’ and ‘Ellagance Purple’), in combination with fluconazole, against the *C. albicans* ATCC 10231 strain, carried out using the checkerboard method. The obtained values of fractional inhibitory concentration indices (FICIs) were interpreted as follows: synergistic when ≤0.5; additive when >0.5 but ≤1; noninteractive when >1 but ≤4; antagonistic when >4 [26]. Individual interactions of lavender flowers essential oils in combination with fluconazole were found to be synergistic (‘Blue River’ FICI-0.136; ‘Ellagance Purple’ FICI-0.264). A synergistic effect was also found in the case of oils isolated from leafy stalks (‘Blue River’ FICI-0.159; ‘Ellagance Purple’ FICI-0.431) and for the combination of fluconazole with one of the main components of lavender oil—linalool (FICI-0.144). The detailed composition of essential oils from flowers and leafy stalks of two studied cultivars of lavender was described in our previous publication [27]. In total, 47 chemical compounds were identified and quantified in the studied essential oils. The contents of the main components of lavender essential oils are shown in Figure 1. The composition of particular essential oils varied significantly depending on the cultivar and the morphological part of the plant. The main components of essential oils from lavender flowers were linalool, linalool acetate, lavandulol acetate, and α-terpineol, but their distribution was significantly different in the studied cultivars. Essential oil from ‘Ellagance Purple’ flowers was characterized by almost twofold higher content of linalool (40.12%) in comparison to ‘Blue River’ flowers (22.51%). On the other hand, ‘Blue River’ flower essential oil contained more esters, linalool acetate (23.16%) and lavandulol acetate (10.73%), than ‘Ellagance Purple’ (16.39% and 4.97%, respectively). The composition of essential oils from leafy stalks was significantly different in comparison to essential oils from lavender flowers. The main components of leafy stalks essential oils were borneol (14.44–16.84%), caryophylene oxide (7.98–8.68%), epi-bicyclosesquiphellandrene (6.46–8.26%), caryophyllene (6.05–7.11%), and eucalyptol (5.66–6.19%). Comparing the main components of essential oils from flowers with those from leafy stalks, the content of linalool was significantly lower (4.90–5.76%), as was the content of linalool acetate (1.28–1.83%) and lavandulol acetate (trace) [27].

## 3. Discussion

In recent years, many scientific publications have presented research on the biological activity of essential oils from medicinal lavender [24,27,28,29,30,31,32,33,34,35]. They exhibit a broad spectrum of antifungal properties, while being less toxic than most chemotherapeutics [15,34,35]. The experiments carried out by other authors showed varied activity of lavender essential oils against the strains of *C. albicans* [31,32,33,35]. A study of Soulaimani et al. [25] demonstrated a synergistic effect of fluconazole combination with essential oils from *Thymus leptobotrys* (FICI = 0.25), *Origanum compactum* (FICI = 0.26), and *Artemisia herba alba* (FICI = 0.27). De Rapper et al. [34] showed an additive effect of *L. angustifolia* oil against *Candida albicans* ATCC 10231 in combination with chloramphenicol (FICI = 1.0) and a lack thereof in combination with nystatin (FICI = 1.14). Göger et al. [36] reported an additive effect for combination of *L. angustifolia* essential oils with ketoconazole against *C. albicans* ATCC 10231 (FICI: 0.53–1.5) and against *C. albicans* clinical strain (FICI: 0.53–1.0). Other authors have experimentally demonstrated differential effects of other essential oils against *C. albicans* strains [14,18,35].

In our study, the evaluation of the effects of lavender oils in combination with fluconazole was carried out using the checkerboard method, comparing the activity of essential oils from two different cultivars and morphological parts of lavender. The calculated FICI values were used to evaluate the effects of their interaction with fluconazole. When analyzing individual combinations of fluconazole with lavender essential oils, positive synergistic effects were recorded in all cases. The results show significant differences between the individual cultivars; ‘Blue River’ lavender essential oil exhibited a higher antifungal activity compared to ‘Ellagance Purple’, while the observed enhancement effect of fluconazole activity was also stronger for ‘Blue River’. In both tested cultivars, essential oils obtained from flowers showed higher activity than essential oils originated from leafy stalks. Interestingly, a similar effect of fluconazole activity enhancement was obtained for essential oil from flowers (FICI = 0.136) and leafy stalks (FICI = 0.159) for the cultivar ‘Blue River’, despite significant differences in their composition. This fact confirms the complexity of the interaction mechanisms in the case of products of natural origin, where even minor components can play an important role in final effect. Importantly, a synergistic effect against the studied *C. albicans* strain was obtained for fluconazole combination with all tested essential oils; thus, it can be concluded that all of them can effectively enhance activity of fluconazole. Nevertheless, observed variations among particular combinations indicate that the differences between essential oils from various cultivars should be taken into consideration in optimizing of final effect. Moreover, not only flowers but also leafy stalks of lavender can be a source of valuable biologically active compounds for pharmacological applications. The activity of essential oils isolated from different cultivars and morphological parts of lavender in combination with fluconazole against *C. albicans* has not been studied so far.

Linalool is one of the main ingredients of lavender flower essential oils. It belongs to the group of unsaturated monoterpene alcohols with an intense aroma of lily of the valley. Hsu et al. [37] described results of studies which confirmed antifungal activity of linalool against *Candida albicans* ATCC 14053. According to Dias et al. [38], linalool is also characterized by a strong biocidal activity against clinical strains of *C. albicans*. Similar results were presented by Zore et al. [39], where antifungal activity of linalool against 39 *C. albicans* clinical strains was confirmed. Researchers in [39] studied the interactions of linalool and five other terpenoids with fluconazole, and a synergistic effect was reported for tested combinations. Synergism of linalool with other azole antibiotics was demonstrated by Ponte et al. [40], who found that *Microsporum* spp. and *Trichophyton* spp. became more susceptible to ketoconazole and itraconazole in the presence of linalool. Our study demonstrated the activity of linalool against *C. albicans* ATCC 10231 strain (MIC = 0.125% *v/v*); moreover, a synergistic effect of its combination with fluconazole (FICI = 0.144) was also shown. The determined antifungal activity of linalool and its interaction with fluconazole proved to be very similar to that of the essential oil obtained from the flowers of the lavender cultivar ‘Blue River’, which contained only 22.51% linalool in its composition. This fact suggests that other components of lavender essential oils also exhibit antifungal activity and enhance the effect of fluconazole, prompting further research in this area.

A useful tool in theoretical prediction of possible interactions of various molecules with active sites of biologically important enzymes is molecular docking and molecular dynamics simulation. Using this method, antifungal activity of the synthetic and natural compounds can be predicted from the visualizations and calculations of their specific interactions with the active sites of the lanosterol 14-α-demethylase, the enzyme responsible for the synthesis of the fungal cell wall. Recent studies of antifungal activity of linalool against the resistant strains of *C. albicans* have shown good correlation between experimental and theoretical studies performed by molecular docking. It was found that linalool probably causes damage of the *C. albicans* cell wall and plasma membrane, what can be explained by its interaction with three important enzymes involved in their biosynthesis, including lanosterol 14-α-demethylase [41]. Other molecular docking studies performed for 60 plant-origin compounds revealed that 48 of them have shown binding affinity with lanosterol 14-α-demethylase [42]. Interesting is that, among them, there are some compounds which are present in lavender essential oils studied by us: caryophyllene oxide (excellent binding activity), geranyl acetate (good binding activity), borneol, carvone, and nerol (medium binding activity). This suggests that the presence of these compounds can also significantly contribute to the effectiveness of lavender essential oils against *C. albicans*, especially in essential oils from lavender leafy stalks, which contained significantly smaller amounts of linalool and higher amounts of caryophyllene oxide and borneol. 

According to Kalemba and Kunicka [43], biological activity depends on the chemical nature of the main components of essential oils and decreases in the following order: phenols > aldehydes > ketones > alcohols > esters > ethers > hydrocarbons. Although the lavender oils used in our study were characterized by a high content of alcohols and esters (linalool, borneol, linalool acetate, and lavandulol acetate), they exhibited high antifungal activity and proved to be very effective fluconazole enhancers.

## 4. Materials and Methods

### 4.1. Isolation and Analysis of Essential Oils

Plant raw material included dried flowers and leafy stalks of two cultivars of *L. angustifolia*: ‘Blue River’ and ‘Ellagance Purple’. The plants were obtained from the Experimental Station of the Department of Horticulture at West Pomeranian University of Technology, Szczecin (Poland). Lavender was cultivated in Szczecin in field conditions and manually collected at a full flowering stage. Each plot area was 6 m in length and consisted of six rows. The distance between the rows was 1 m with 50 cm intra-row spaces. The field was set up on typical black earth soil of quality class III, with a pH of 6.5. After collection, lavender was air-dried at room temperature for 4 weeks in a shaded laboratory room. Directly before the studies, the dried raw material was divided into two groups: flowers and leafy stalks. Flowers were then crushed in a mortar while leafy stalks cut into ca. 1 cm pieces and ground in a laboratory mill. Next, the weighed sample (20.00 g) was placed in a 1000 mL round-bottom flask, immersed in 400 mL of distilled water, and connected to a Deryng apparatus. The hydrodistillation process was performed for 3 h, and the obtained oils were dried over anhydrous sodium sulfate. For this purpose, 1.0 mL of methylene chloride and desiccant were added to the vial with essential oil. After storage in the refrigerator for 24 h, the drying agent was removed by filtration, and the solvent was evaporated to obtain a pure essential oil. Yields of essential oils obtained from various cultivars and morphological parts of lavender ranged from 0.48% to 1.92%. The highest yields were obtained for essential oils isolated from flowers: ‘Ellagance Purple’, 1.92%; ‘Blue River’, 1.52%. In the case of leafy stalks, lower yields were obtained: ‘Ellagance Purple’, 0.65%; ‘Blue River’, 0.48%. The composition of all essential oils was studied using gas chromatography with the mass selective detector (GC–MS) method. The chromatographic analyses were performed using an Agilent 6890N gas chromatograph with a 5973N mass selective detector and a 7683 Series Injector, after dissolving essential oils (0.02 mL) in 1.0 mL of acetone (p.a.). Samples (3 μL) were injected via split (10:1) to an HP-5MSI column (30 m length, 0.25 mm ID, 0.25 μm film thickness) for the separation of particular compounds in optimized conditions. The column temperature was programmed from 60 °C (hold 3 min), with a ramp rate of 5 °C/min to a final temperature of 300 °C (hold 1 min). The temperature of the injector was set at 250 °C, of the MS source was set at 230 °C, and of the MS quad was set at 150 °C; helium with a flow rate of 1.2 mL/min was used as a carrier gas. The identification of the main compounds of the essential oils were carried out based on mass spectra and retention indices (Supplementary materials). In order to confirm the identification, the retention indices of the compounds were calculated and compared with the literature [44]. The obtained results of the chemical composition analysis were statistically analyzed using analysis of variance for one-way cross-classification, separately for each compound and morphological part, evaluating the significance of differences between cultivars of lavender with Student’s t-test calculated at a confidence level of *p* ≤ 0.05. Statistical analysis was performed using the PQStat v. 1.6.2 package (PQStat Sofware 1.6.2, Poznań, Poland).

### 4.2. Microdilution Checkerboard Method

Minimum inhibitory concentration (MIC) microdilution assays were performed to determine the antifungal activity of lavender essential oils, linalool (Merck, Darmstadt, Germany), and fluconazole (2 mg/mL; RT Pharma, Lamarie). The MIC values were considered the concentrations at which there was no visible turbidity and growth of *C. albicans*, due to the fungicidal effect. The obtained results were verified according to CLSI M27 recommendations [45]. Dimethyl sulfoxide—DMSO (POCH, Gliwice, Poland) was used as a solvent to prepare the desired dilutions of lavender essential oils. In the performed assays, the *C. albicans* ATCC 10231 strain of the American Type Culture Collection was used. The fungi were inoculated by reductive culture into a Columbia Blood agar with 5% of sheep blood (Oxoid, UK) medium and incubated at 37 °C for 48 h. After this time, 2–5 typical colonies of the working strain culture were collected and suspended in an isotonic and sterile 0.85% solution of NaCl. Next, measurements of optical density were performed using density meter (with measurement deviation of ±0.1, in the range of 0.00–3.00 according to McFarland’s scale) causing turbidity of 0.5 MF, which corresponded to the average quantity of 1.5 × 10^8^ CFU/mL. Sterile, divided, and individually packed 96-wells polystyrene titration plates with flat bottoms were used in the studies. A series of twofold dilutions of each antimicrobial agent were prepared in a ready-to-use Roswell Park Memorial Institute (RPMI) 1640 Culture Medium with glutamine and phenol red (Gibco, Thermo Fisher Scientific, Waltham, MA USA) ranging from 0.005 to 200 µg/mL for fluconazole, while, for lavender essential oils and linalool, the concentration gradients were 0.005–5% *v/v*. At the next stage, 0.005 mL of *C. albicans* suspension was added to each well (at final concentration in the well of 10^5^ CFU/mL). To exclude an inhibitory effect of DMSO and possible contaminations, both positive and negative control assays were also performed. The resulting checkerboard contained a combination of a concentration gradient of antifungal agents, wherein wells that contained the highest concentration of each agent were located at opposite corners of a plate. After sealing, the plates were incubated at 35 ± 1 °C for 48 h. After incubation, MIC was defined as the lowest concentration that did not result in any visible growth of the fungal strains compared to their growth in the control wells [46]. All determinations of MIC were performed in triplicate. MIC data of the essential oils and fluconazole were converted into the fractional inhibitory concentration (FIC), defined as the ratio of the concentration of the antimicrobial in an inhibitory concentration with a second compound to the concentration of the antimicrobial by itself [26]. The checkerboard procedure in the combination assays described by Rosato et al. was applied to evaluate the type of interactions of the lavender essential oils with fluconazole [20]. The combinations of essential oils obtained from various cultivars and morphological parts of lavender with fluconazole were analyzed by calculating the FIC index (FICI) using the following formulas:

(1) $$\mathrm{FIC}\;\mathrm{of}\;\mathrm{essential}\;\mathrm{oil}=\frac{\mathrm{MIC}\;\mathrm{value}\;\mathrm{of}\;\mathrm{essential}\;\mathrm{oil}\;\mathrm{combined}\;\mathrm{with}\;\mathrm{fluconazole}}{\mathrm{MIC}\;\mathrm{value}\;\mathrm{of}\;\mathrm{essential}\;\mathrm{oil}\;\mathrm{alone}},$$

(2) $$\mathrm{FIC}\;\mathrm{of}\;\mathrm{fluconazole}=\frac{\mathrm{MIC}\;\mathrm{value}\;\mathrm{of}\;\mathrm{fluconazole}\;\mathrm{combined}\;\mathrm{with}\;\mathrm{essential}\;\mathrm{oil}\;}{\mathrm{MIC}\;\mathrm{value}\;\mathrm{of}\;\mathrm{fluconazole}\;\mathrm{alone}},$$

(3) FICI=FIC value of essential oil+FIC value of fluconazole.

In order to evaluate the interaction of linalool with fluconazole, analogous studies were performed, and FIC and FICI values were calculated by replacing essential oil with linalool in the above formulas. The obtained FICI values were interpreted as follows: synergistic when ≤0.5; additive when >0.5 but ≤1; noninteractive when >1 but ≤4; antagonistic when >4 [26].

## 5. Conclusions

The tested essential oils isolated from two cultivars of *L. angustifolia*, ‘Blue River’ and ‘Ellagance Purple’, represent a promising group of substances of natural origin with antifungal activity. A very interesting observation regarding the activity of the studied natural products was that, despite the observed differences in composition and activity, all studied essential oils, which also originated from leafy stalks, showed a significant synergistic effect in combination with fluconazole against the *C. albicans* strain. Considering the demonstrated synergy of the tested essential oils with fluconazole, further research is necessary to confirm the effectiveness of these combinations through in vivo studies.

## Figures and Tables

**Table 1:** Lavender essential oils and linalool activity in combination with fluconazole against *C. albicans* ATCC 10231 strain.

Antifungal Agent	MIC_0_	MIC_c_	FIC	FICI	Type of Interaction
Essential oils from lavender flowers in combination with fluconazole					
‘Blue River’ (% *v/v*)	0.125	0.016	0.128	0.136	Synergistic
Fluconazole (μg/mL)	20	0.160	0.008		
‘Ellagance Purple’ (% *v/v*)	0.250	0.062	0.248	0.264	Synergistic
Fluconazole (μg/mL)	20	0.320	0.016		
Essential oils from lavender leafy stalks in combination with fluconazole					
‘Blue River’ (% *v/v*)	0.250	0.032	0.128	0.159	Synergistic
Fluconazole (μg/mL)	20	0.625	0.031		
‘Ellagance Purple’ (% *v/v*)	0.625	0.250	0.400	0.431	Synergistic
Fluconazole (μg/mL)	20	0.625	0.031		
Linalool in combination with fluconazole					
Linalool (% *v/v*)	0.125	0.016	0.128	0.144	Synergistic
Fluconazole (μg/mL)	20	0.320	0.016		

MIC—minimal inhibitory concentration; FIC—fractional inhibitory concentration; FICI—fractional inhibitory concentration index; MIC_0_ = MIC of an individual sample alone; MICc = MIC of an individual sample of the most effective combination; FIC of essential oil or linalool = MIC of essential oil or linalool in combination with fluconazole/MIC of essential oil or linalool alone; FIC of fluconazole = MIC of fluconazole in combination with essential oil or linalool/MIC of fluconazole alone; FICI = FIC of essential oil or linalool + FIC of fluconazole. Interpretation of results: FICI ≤0.5, synergistic; FICI >0.5–1.0, additive; FICI >1.0–4.0, no interaction; FICI >4.0, antagonistic [26].

## Data Availability

Not applicable.

## Supplementary Materials

The following supporting information can be downloaded at https://www.mdpi.com/article/10.3390/molecules28031176/s1: Table S1. Retention parameters of compounds identified in essential oils from flowers and leafy stalks of *Lavandula angustifolia* cultivars ‘Blue River’ and ‘Ellagance Purple’, obtained by GC–MS; Figure S1. Chromatogram of essential oil from flowers of ‘Blue River’ cultivar of *Lavandula angustifolia*; Figure S2. Chromatogram of essential oil from flowers of ‘Ellagance Purple’ cultivar of *Lavandula angustifolia*; Figure S3. Chromatogram of essential oil from leafy stalks of ‘Blue River’ cultivar of *Lavandula angustifolia*; Figure S4. Chromatogram of essential oil from leafy stalks of ‘Ellagance Purple’ cultivar of *Lavandula angustifolia*; Figure S5. Mass spectrum of eucalyptol present in *Lavandula angustifolia* essential oils, compared with eucalyptol standard mass spectrum from NIST 02 library; Figure S6. Mass spectrum of linalool present in *Lavandula angustifolia* essential oils, compared with linalool standard mass spectrum from NIST 02 library; Figure S7. Mass spectrum of borneol present in *Lavandula angustifolia* essential oils, compared with borneol standard mass spectrum from NIST 02 library; Figure S8. Mass spectrum of linalool acetate present in *Lavandula angustifolia* essential oils, compared with linalool acetate standard mass spectrum from NIST 02 library; Figure S9: Mass spectrum of lavandulol acetate present in *Lavandula angustifolia* essential oils, compared with lavandulol acetate standard mass spectrum from NIST 02 library; Figure S10. Mass spectrum of caryophyllene present in *Lavandula angustifolia* essential oils, compared with caryophyllene standard mass spectrum from NIST 02 library; Figure S11. Mass spectrum of caryophyllene oxide present in *Lavandula angustifolia* essential oils, compared with caryophyllene oxide standard mass spectrum from NIST 02 library; Figure S12. Chromatogram of linalool standard; Figure S13. Mass spectrum of linalool, compared with linalool standard mass spectrum from NIST 02 library.

## Sample Availability

Samples of essential oils are available from the authors.
